# Insecticidal Activity of Essential Oils against Mealybug Pests (Hemiptera: Pseudococcidae): A Systematic Review and Meta-Analysis

**DOI:** 10.3390/plants12010109

**Published:** 2022-12-26

**Authors:** Miriam del Valle Avila, Fernanda Achimón, Vanessa Daniela Brito, Ramiro Aguilar, Romina Paola Pizzolitto, María Paula Zunino, María Laura Peschiutta

**Affiliations:** 1 Cátedra de Química Orgánica, Facultad de Ciencias Exactas, Físicas y Naturales, Universidad Nacional de Córdoba, Av. Vélez Sarsfield 1611, Córdoba X5016GCA, Argentina; 2 Instituto Multidisciplinario de Biología Vegetal (IMBIV), CONICET-Universidad Nacional de Córdoba, Av. Vélez Sarsfield 1611, Córdoba X5016GCA, Argentina

**Keywords:** Pseudococcidae, toxicity, essential oils, insecticides, meta-analysis

## Abstract

Most mealybugs of the Pseudococcidae family are important pests of agriculture and ornamental gardens. Our aim was to perform a review and meta-analysis on 14 published scientific articles on the insecticidal activity of essential oils (EOs) against mealybug species of the Pseudococcidae family. Data on (1) species, genus, families, and plant parts from which the EO was extracted; (2) the main compounds of each EO; (3) the highest and lowest concentrations tested; and (4) the application method used for the toxicological studies was collected from each study. The metafor package (R software) was used to perform a three-level random effects meta-analysis. The families Lamiaceae, Rutaceae, Myrtaceae, Zingiberaceae and Euphorbiaceae and the genera *Citrus*, *Cymbopogon*, *Syzygium*, *Cinnamomum* and *Jatropha* were the most used among the studies. According to the results from the meta-analyses, 13 out of 24 genera analyzed were effective against mealybugs. All methods were effective, but fumigation and indirect contact were the most frequently used methodologies. The results obtained from the present review and meta-analysis could be used for the potential development of natural biopesticide formulations against mealybugs belonging to the Pseudococcidae family.

## 1. Introduction

The commonly known mealybugs or coccids include all members of the Coccoidea superfamily (Hemiptera), which is composed of 28 families [1]. Among them, one of the most important family is Pseudococcidae, which comprises insects characterized by a soft, oval body, mostly covered by a floury layer and waxy secretions, with lateral and caudal extensions in varying length according to the species [1,2]. Most mealybugs of the Pseudococcidae family are major pests in agriculture and ornamental gardens [3,4] and can be found infesting the leaves, branches and roots of their host plant [1,2]. They can feed on plants such as grapevine, coffee, pineapple, cotton and citrus, among other fruit plants. In addition, they can also infest palm trees, cacti and succulents, and different ornamental plants [5,6,7].

Mealybug species that feed on fruit trees negatively affect fruit production by sucking sap from the phloem, excreting large amounts of sugar and water as a sugary, carbohydrate-rich substance known as honeydew. This substance causes severe secondary damage, as it promotes the growth of sooty mold (black fungus), which decreases photosynthesis and affects the development of the host plant [8]. Fruits stained with sooty mold or suspected of containing mealybugs are rejected when exported, due to strict phytosanitary regulations; and these economic losses due to infestations by mealybugs have increased dramatically in recent years [6].

The control of these phytophagous insects in agroecosystems is difficult due to their small body size and cryptic nature [9]. Currently, the main control is based on the application of synthetic pesticides [6,8]. The sustained use of synthetic pesticides contributes to the crisis in agriculture that affects ecosystems, natural resources, as well as the health of rural communities and urban consumers [10,11], in addition to generating resistance in pests [12]. Consequently, these negative effects have highlighted the need to develop new eco-friendly effective insecticides. In this context, several studies have proposed certain botanical products as safer natural alternatives to synthetic insecticides [13,14,15,16], such as essential oils (EOs) derived from aromatic plants [17,18]. Essential oils consist of complex mixtures of approximately 20–60 different volatile organic compounds (VOCs), of which only two or three are present at high concentrations, while the others are considered minor constituents [19,20]. The VOC profile of EOs can vary not only between species, but also according to the part of the plant used for EO extraction [20,21]. There are also important differences in the chemical composition and bioactivity of a single EO depending on the geographical distribution, harvest time, growth conditions, and developmental stage of the plant used to obtain the EO as well as the extraction methods [22]. In addition, the same EO can exert different effects due to the variety of experimental procedures available to evaluate insecticidal activity, such as application method [13,23,24]. The EOs can be applied directly to the insect by spraying or through topical application, or indirectly by spraying or immersing the substrate; or by fumigation (EO vapor in the air) [25,26,27].

Despite the well-known advantages of using EOs for agricultural pest management, so far, the state of the art and the perspective of EO development for pseudococcid control have not been reviewed. Therefore, the aim of the present study was to analyze the insecticidal effect (mortality) of plant EOs against mealybugs (Pseudococcidae) through a systematic review and meta-analysis.

## 2. Results

**Figure 1:**
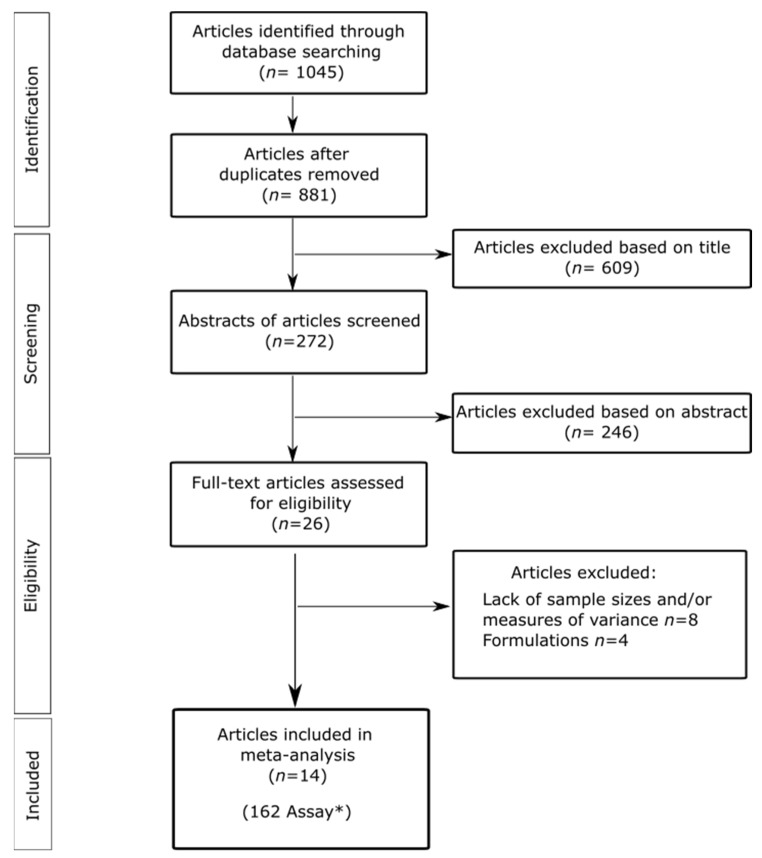
Flowchart for the selection of articles according to the criteria established for systematic reviews and meta-analysis (PRISMA). * Each trial for the meta-analysis was defined for a given plant EO/exposure time/max-min concentration of EO/mealybug species tested/development stage of insect/method of application.

Figure 1 presents a summary of the literature search through different multidisciplinary databases. The initial search returned a total of 1045 articles. After removing duplicates (*n* = 164), 881 were recovered; next, 609 were excluded based on their titles, followed by 246 articles being excluded based on their abstract. Then, the full text of the 26 resulting articles was evaluated. Twelve studies did not meet the selection criteria: 4 articles evaluated insecticidal formulations and 8 articles lacked information regarding sample sizes and/or variance measures or only reported LC_50_. Finally, 14 studies (162 assays) met the inclusion criteria and were included in the analyses. The selected articles and the main compounds of the EOs are presented in Table 1.

Lamiaceae (18.52%), Rutaceae (14.81%), Euphorbiaceae (11.11%), Myrtaceae (11.11%), and Zingiberaceae (11.11%) were the families most frequently used in the studies, while the rest of the families accounted for 33.34%. The genera *Citrus* (14.81%), *Cymbopogon* (9.26%), *Cinnamomum* (7.41%), *Jatropha* (7.41%), and *Syzygium* (7.41%) were the most used in the studies, while the remaining genera accounted for 53.70%. *Cymbopogon citratus* (7.41%), *Jatropha curcas* (7.41%), and *Syzygium aromaticum* (7.41%) were the most frequently used species against mealybugs while the rest of the species accounted for 77.77%. On the other hand, the part of the plant more frequently employed for the extraction of EOs were the leaves (27.78%) and seeds (20.37%), followed by fruit peels (12.96%), aerial parts (11.11%), roots (7.41%), and buds (3.70%). Furthermore, it should be noted that 16.67% of the studies did not report from which part of the plant the EOs were extracted.

The studies conducted using the fumigant method (46.30%), where the EOs saturated the atmosphere of the containers containing the mealybugs without direct contact with them, were the most frequently represented among the studies. Less frequent were those studies carried out by indirect contact (31.48%) either by spraying the substrate with EOs or submerging the substrate into EOs. This substrate was generally a filter paper or food (coffee leaves, conil, papaya, *Citrus reticulata* branches and *Hibiscus rosa-sinensis* leaves). On the other hand, studies conducted with direct contact methodology where the EOs were sprayed directly on the insect were less represented (22.22%).

The mealybug species of the Pseudococcidae family that were the most frequently used to test the insecticidal effect of EOs were *Pseudococcus jackbeardsleyi* (33.33%), followed by *Maconellicoccus hirsutus* (22.22%), and *Planococcus citri* (11.11%) while the rest of the species accounted for 33.34% of the total studies. The mealybug development stage more represented was the nymphal stage (53.70%), followed by adults (38.89%), while the remaining trials (7.41%) did not report the development stage.

**Figure 2:**
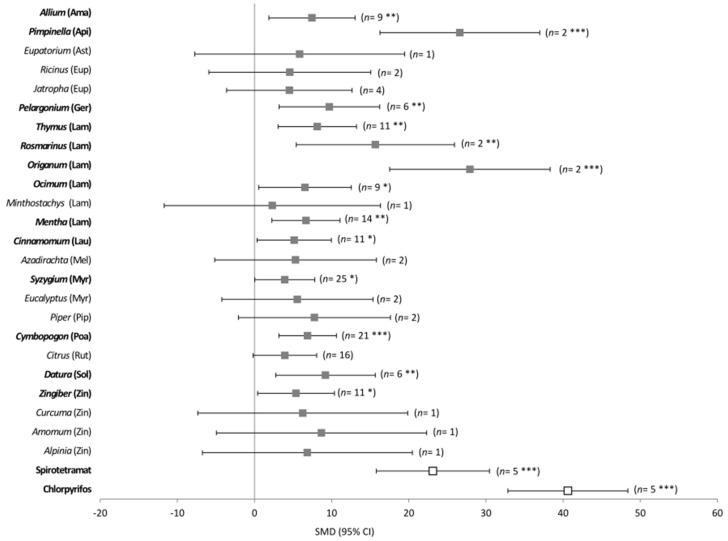
Meta-analysis of the insecticidal effect of EOs against mealybugs of the Pseudococcidae family using plant genera as a moderating variable. Spirotetramat and chlorpyrifos are synthetic insecticides (white squares) used to compare their activity with the EOs ones (black squares). Abbreviations are as follows: *Amaryllidaceae* (Ama), *Apiaceae* (Api), *Asteraceae* (Ast), *Euphorbiaceae* (Eup), *Lamiaceae* (Lam), *Lauraceae* (Lau), *Myrtaceae* (Myr), *Poaceae* (Poa), *Rutaceae* (Rut), *Geraniaceae* (Ger), *Meliaceae* (Mel), *Piperaceae* (Pip), *Solanaceae* (Sol), *Zingiberaceae* (Zin). SMD: standardized mean difference; CI: confidence interval. The EOs from plant genera with significant effects are shown in bold (their mean value is different from zero). *** *p* < 0.001, ** *p* < 0.01, and * *p* < 0.05.

According to the meta-analyses conducted, the genera *Allium* (Amaryllidaceae), *Pimpinella* (Apiaceae), *Pelargonium* (Geraniaceae), *Mentha* (Lamiaceae), *Ocimum* (Lamiaceae), *Origanum* (Lamiaceae), *Rosmarinus* (Lamiaceae), *Thymus* (Lamiaceae), *Cinnamomum* (Lauraceae), *Syzygium* (Myrtaceae), *Cymbopogon* (Poaceae), *Datura* (Solanaceae), and *Zingiber* (Zingiberaceae) showed mean effects very different from the rest of the genera, with *Origanum* and *Pimpinella* being the ones with higher effects, similar to those of the synthetic insecticides chlorpyrifos and spirotetramat (QM = 110.6218, df = 25, *p* < 0.0001; Figure 2).

**Figure 3:**
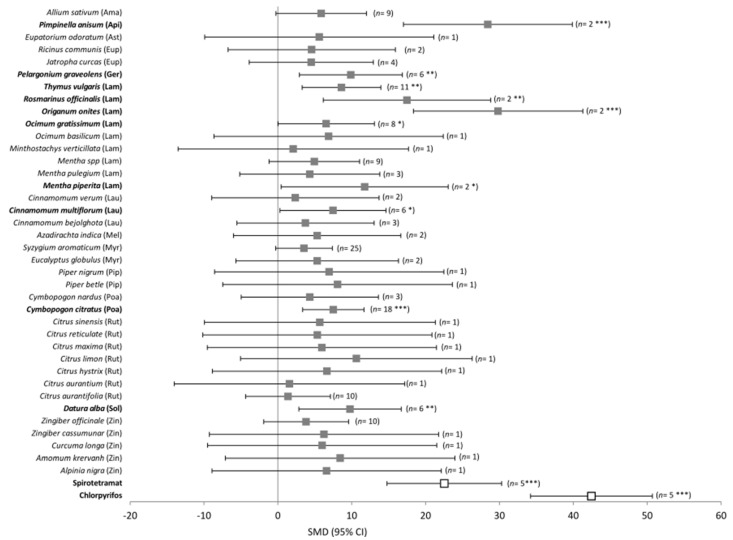
Meta-analysis of the insecticidal effect of EOs against mealybugs of the Pseudococcidae family using the plant species as a moderating variable. Spirotetramat and chlorpyrifos are synthetic insecticides (white squares) used to compare their activity with the EOs ones (black squares) Abbreviations as in Figure 2. SMD: standardized mean difference; CI: confidence interval. The EOs from plant species with significant effects are shown in bold. *** *p* < 0.001, ** *p* < 0.01, and * *p* < 0.05.

The EOs of *Pimpinella anisum* (Apiaceae), *Pelargonium graveolens* (Geranaceae), *Thymus vulgaris* (Lamiaceae), *Rosmarinus officinalis* (Lamiaceae), *Ocimum gratissimum* (Lamiaceae), *Mentha piperita* (Lamiaceae), *Cinnamomum multiflorum* (Lauraceae), *Origanum onites* (Lamiaceae), *Cymbopogon citratus* (Poaceae), and *Datura alba* (Solanaceae) showed insecticidal effect against mealybugs, with *P. anisum* and *O. onites* EOs reporting insecticidal activities similar to those of chlorpyrifos and spirotetramat (QM = 118.68, df = 39, *p* < 0.0001, Figure 3). 

**Figure 4:**
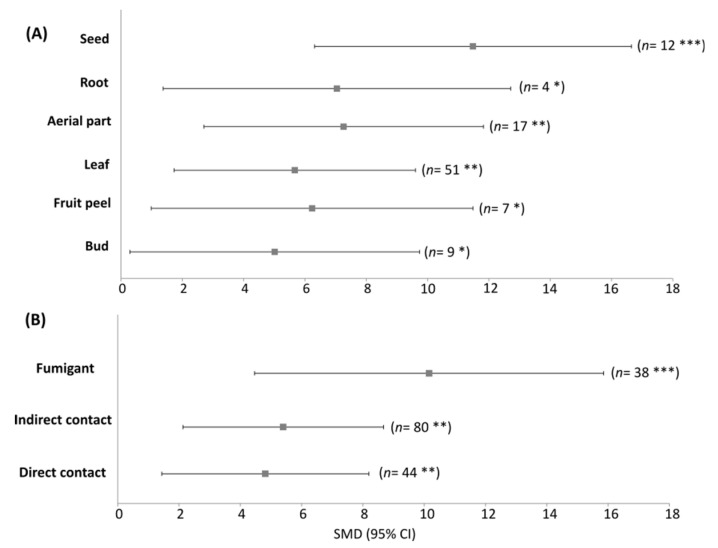
Meta-analysis of the insecticidal effect of EOs against mealybugs of the Pseudococcidae family using plant parts (**A**) and application method (**B**) as moderating variables. The EOs from plant parts and application method with significant effects are shown in bold. *** *p* < 0.001, ** *p* < 0.01, and * *p* < 0.05.

In addition, the effects of EOs extracted from the different tissues (roots, seeds, fruit peels, leaves and buds, and aerial parts) were similarly effective as insecticides (QM = 7.46, df = 5, *p* = 0.19; Figure 4a). Also, all application methods had similar effects as insecticides, with no statistically significant differences among them (QM = 2.53, df = 2, *p* = 0.28, Figure 4b).

## 3. Discussion

Plant EOs have been the subject of investigation by many disciplines due to their wide range of bioactivities including antimicrobial and insecticidal, as well as therapeutic and medicinal effects [28]. In this review, we found that Lamiaceae, Rutaceae, Myrtaceae, Euphorbiaceae, and Zingiberaceae were the most frequently evaluated families against mealybugs. Our results are in agreement with other authors who found that these EOs were more common in mortality tests against mosquitoes [29] and stored product insects [17,30,31]. Essential oils from *Citrus* (Rutaceae), *Cymbopogon* (Poaceae), *Cinnamomum* (Lauraceae), *Jatropha* (Euphorbiaceae), and *Syzygium* (Myrtaceae) were the most represented. The classical EO extraction method is based on the steam distillation apparatus (Clevenger) developed in 1928. Today, this method has been adapted and extended for industrial production. Steam distillation requires large vessels due to the low yield (typically < 1%) of the biomass and is expensive due to the high temperatures required for distillation. *Citrus* peel is an exception due to the large quantities of EOs that can be obtained cheaply by cold pressing and conventional distillation [17]. The ease of obtaining this EO, in addition to its effectiveness (high content of limonene) make the *Citrus* genus one of the most chosen for laboratory experiments. However, many species other than *Citrus* spp. were also widely used in studies, generally due to their widespread distribution, low cost, and ease of availability on the market. One of these species was the clove (*Syzygium aromaticum*, Myrtaceae), a valuable spices that has been used for centuries as a food preservative and for medicinal purposes [32]. Another widely used species was lemongrass (*Cymbopogon citratus*, Poaceae), which is distributed worldwide and is cultivated mainly for its EO, which is of considerable commercial importance due to its use in the manufacture of fragrances, flavors, perfumery, cosmetics, detergents, and pharmaceuticals. In addition, the non-edible EO of *Jatropha curcas* is currently considered as an important raw material for biodiesel production [33,34]. According to the results of the meta-analyses, 13 out of the 24 genera analyzed were effective against mealybugs. For example, a recent publication found that adults of *P. ficus* were more susceptible to EO from *Cymbopogon citratus* (LC_90_ = 0.01 µL/cm^2^), in relation to EOs from *Pelargonium graveolens* (LC_90_ = 0.14 µL/cm^2^) and *Mentha piperita* (LC_90_ = 0.34 µL/cm^2^) [35]. Erdemir and Erler [36] compared fumigant effects of several EOs on *Planococcus citri* after 24 h of exposure and found the following order of toxicity: *Origanum onites* (LC_50_ = 1.17 μL/L air) > *Thymus vulgaris* (LC_50_ = 1.44 μL/L air) > *Pimpinella anisum* (LC_50_ = 1.57 μL/L air) > *Rosmarinus officinalis* (LC_50_ = 2.64 μL/L air) > *Mentha piperita* (LC_50_ = 3.27 μL/L air). Furthermore, Ghafoor, et al. [37] found that *Datura alba* EO (LC_50_ = 2.16 and 0.80% *v*/*v*) was more effective against *Drosicha mangiferae* than EOs from *Cymbopogon citratus* (LC_50_ = 12.25 and 1.27% *v*/*v*) and *Syzygium aromaticum* (LC_50_ = 6.31 and 0.90% *v*/*v*) at 48 and 72 h, respectively. Most plant EOs showed similar effectiveness to spirotetramat, which is highly effective against sap-sucking scale insects and is widely used in the field to combat mealybug pests [38]. However, only *P. anisum* and *O. onites* EOs showed insecticidal activity similar to that of chlorpyrifos, which is considered more toxic and harmful than spirotetramat. Chlorpyrifos has been regarded among the most commonly applied and effective insecticide against scale insects (mealybugs and armored scales) in the field [9,38,39,40]. Although chlorpyrifos generally produce high toxicity against mealybugs, this synthetic insecticide presents some limitations, for example, adverse effects on non-target natural enemies and insect pollinators, in addition to development of resistance in scale insects [9,41,42]. Therefore, the use of plant EOs such as *P. anisum* and *O. onites* should be considered for controlling mealybugs over the synthetic insecticides when implementing eco-friendly integrated pest management programs.

Although the toxicity and repellency of plant EOs and their terpenoid constituents have been long recognized, the exact biochemistry and mechanisms of action remain to be fully understood, particularly in mealybugs. Knowledge on the chemical properties of EO compounds is necessary to determine the safety and economy of their use in agriculture. Insecticides of natural origin can affect the physiology of insects in different pathways and receptor sites. Essential oils and their constituents affect many biochemical processes. They can specifically produce neurological or endocrinological imbalances in insects; for example, they can act as insect growth regulators, disrupting the normal process of morphogenesis [43]. Acetylcholinesterase (AChE) plays a role in cholinergic synapses which is crucial for insects and higher animals. The inhibition of AChE, one of the most important modes of action of VOCs, causes the accumulation of acetylcholine at the synapse site; the postsynaptic membrane is permanently stimulated, resulting in ataxia, loss of coordination in the nervous and neuromuscular systems and eventually death [44]. Recent studies by Brahmi, et al. [35] found that the EO from *Cymbopogon citratus*, an effective species against mealybugs, affects the nervous system of adult *Planococcus ficus*, which was evidenced by a significant inhibition of AChE activity. The main components of this EO, citral and limonene, are known as AChE inhibitors in electric eel (freshwater fish) and rice weevil (insect), respectively [45,46]. Limonene also has the ability to degrade lipids from the cuticle of the insect exoskeleton [47]. It has been reported in several studies that eucalyptol, one of the main components of EO from *Rosmarinus officinalis* that were effective against mealybugs, showed strong AChE inhibitory activity in different insects [45,48,49]. A similar pattern was obtained with EOs from *Origanum onites* and *Thymus vulgaris* with terpinen-4-ol as a constituent of their EOs [45]. The EOs of two species that were effective against mealybugs, *Datura alba* and *Origanum onites*, present carvacrol as one of their main compounds. This compound is also known as an AChE inhibitor in some insects such as *Drosophila melanogaster* [50]. Furthermore, carvacrol can interact with the octopamine receptor by altering the conformation and increasing the affinity for endogenous G-protein in the American cockroach [51]. The genus *Datura* and the species *Ocimum gratissimum* also have thymol as their main component [52]. This compound can act at the level of the GABA system, blocking GABA channels, thus reducing neuronal inhibition and leading to hyperexcitation of the central nervous system, seizures, and death. Another mode of action of thymol is its interaction with the octopamine receptor [43]. Similarly, p-cymene and trans-anethole, the main compounds of *Thymus vulgaris* and *Ocimum gratissimum*, and *Pimpinella anisum*, respectively, were also found as strong antagonists of the octopamine receptor [45].

The bioactivity of an EO is usually attributable to its major component; however, the general activity of the EO is usually explained by the sum of the activities of the individual components, showing additive, synergistic, or antagonistic effects [28]. For example, both isomers (thymol and carvacrol) could synergize in *Datura alba* EO and increase their insecticidal activity in relation to their individual effect, similar to what was reported for *Spodoptera littoralis* [53].

The results from the present study showed that the EOs extracted from all plant parts were effective against mealybugs, with leaves and seeds being the most frequently used among the studies. The variability of the active compounds in these EOs can be attributed to several factors, such as climatic conditions, type of water and soil, harvest time, part of the plant, age of the plant, type of plant sample used (fresh or dry), geographic factors (location), genetic factors (chemotype), and extraction method [45,54,55]. The application methods of EOs can also play an important role in their bioactivity. All methods were effective against these insects, with fumigation being the most frequently used against mealybugs. The main routes of insecticide entry into the arthropod body include the oral-digestive route (digestive tract), the dermal contact route (tarsi, antennae or the entire surface of the cuticle and intersegmental membranes), and the respiratory-inhalation route (spiracle and tracheal system of insects) [56]. Traditional contact insecticide treatments against mealybug populations show limited efficacy in reducing the density of mealybug eggs, nymphs, and adults. This could be explained by their cryptic behavior (many reside hidden under the trunk bark) and the waxy excretions that coat their bodies, which could hinder the ability of these insecticides to achieve full contact with the pest [9]. On the other hand, EOs can penetrate the waxy layer of these insects since these compounds are quite lipophilic, so they can quickly enter and interfere with physiological functions [57]. Fumigation, conversely, is a method that allows a more homogeneous distribution of EOs and has a high ability to move through the insect cuticle or enter through its respiratory system [23].

The present work reviewed the insecticidal effect of EOs against mealybugs of the Pseudococcidae family. A wide spectrum of modes of action was described for these EOs, which is an important feature to prevent the development of pest resistant populations. Essential oils are positioned as excellent botanical insecticides to combat mealybug pests, because these insects develop a waxy layer that makes them less susceptible to synthetic insecticides [26]. Additionally, EOs have low toxicity for mammals and humans and have low persistence in the environment. The results obtained from this review and meta-analyses could be used for the development of future eco-friendly biopesticide formulations against mealybugs of the Pseudococcidae family.

## 4. Materials and Methods

The systematic review and meta-analysis were performed according to the Preferred Reporting Items for Systematic Reviews and Meta-Analysis (PRISMA) criteria [58]. The studies were obtained from eleven electronic databases: Scopus, ScienceDirect, SciELO, JSTOR, Wiley Online Library, Network of Scientific Journals of Latin America and the Caribbean, Spain and Portugal, Cambridge University Press, BioOne, SpringerLink, Taylor & Francis and Academic Google. We used the search construct “(Pseudococcidae) AND (“essential oils” OR “essential oil”) AND (mortality)” to find primary literature on insecticidal activity of EOs on mealybugs. The collection of primary studies was created using the Zotero bibliographic manager [59], and duplicate records were deleted. Then, the quality of the remaining articles was evaluated for the meta-analysis. Papers were included only if they met the following criteria: (1) full-text articles published from 2000 (1 January 2000) (according to the criteria established by Monsreal-Ceballos, et al. [60]) to 31 October 2022; (2) studies reported adult or nymphal mortality; and (3) studies provided means, sample sizes, and measures of variance (standard deviation or standard error) for at least two EO concentrations. The study selection was conducted first by title, then by abstract and finally by reading the complete work. Two reviewers independently performed eligibility assessment and data extraction. Disagreements were arbitrated by a third reviewer and then resolved by consensus. From each study, the following information was collected: (1) the species, genera, families and parts of the plants from which the EO was obtained; (2) highest and lowest concentrations tested; (3) the type of application or method used for the toxicological studies, including contact (direct or indirect) or fumigant; (4) the three main compounds of each EO; and (5) species and developmental stage of the mealybug tested (these last two variables were only included for the systematic review). When a study did not report the main compounds of the EOs, the VOC profile was obtained from other literature articles (Table 1).

### Statistical Analysis

The standardized mean difference (SMD) between high doses and low doses of EOs and the corresponding sample variance for each study were calculated, according to the following formula: SMD = (m1i − m2i)/sdpi, where m1i and m2i are the observed means of the two groups (high dose and low dose, respectively), sdpi = sqrt(((n1i − 1) × sd1i^2^ + (n2i − 1) × sd2i^2^)/(n1i + n2i − 2)) is the combined standard deviation of the two groups, where sd1i and sd2i are the observed standard deviations, and n1i and n2i are the number of individuals in each group.

Values of standard error were transformed to standard deviation according to the equation: SD = SE √ n, where SD is the standard deviation, SE is the standard error, and n is the sample size. Several of the recovered articles presented more than one effect (e.g., effects for different plant species in the same article). For that reason, for each calculated effect we included the study identity as a nested random factor. Thus, we incorporated a new level of variation that included the dependence of the effects obtained from the same study [95]. The rma.mv function from the metafor package that is invoked in R (version 3.2.2) was used to perform a three-level random-effects meta-analysis [95,96,97]. Species, genera and plant parts, and application method were included as moderators. The QM statistic that measures the variance between the groups being compared is reported. The EO insecticidal activity of plant species and genera were compared with two synthetic insecticides, spirotetramat (a tetramic acid derivative belonging to the main chemical group Inhibitors of acetyl CoA carboxylase [98]), applied at its field recommended (registered) dose (120 mL/hL), and chlorpyrifos (an organophosphate belonging to the main chemical group Acetylcholinesterase inhibitors [98]), applied at its field recommended (registered) dose (100 mL/hL) [38].

## Figures and Tables

**Table 1:** Plant EOs evaluated for their toxicity against mealybugs from the Pseudococcidae family.

Mealybug Species	Plant Species	Plant Genus	Plant Family	EO Main Compounds (%)	Ref.
*Planococcus citri*	*Mentha pulegium*	*Mentha*	Lamiaceae	pulegone (40.5), menthone (26.3), isomenthone (5.0) [38]	[38]
*Planococcus minor*	*Syzygium aromaticum*	*Syzygium*	Myrtaceae	eugenol (80.0), eugenyl acetate (5.01), β-caryophyllene (2.27) [61]	[62]
*Formicococcus njalensis*	*Ocimum gratissimum*	*Ocimum*	Lamiaceae	p-cymene (37.0), thymol (18.7), α-thujene (7.4) [34]	[63]
*Maconellicoccus hirsutus*	*Allium sativum*	*Allium*	Amaryllidaceae	diallyl-trisulfide (37.3–45.9), diallyl-disulfude (17.5–29.1), methyl-allyl-trisulfide (7.7–10.4) [64,65]	[27]
*Maconellicoccus hirsutus*	*Mentha* sp.	*Mentha*	Lamiaceae	menthol (3.3–81.3), piperitenone-oxide (10.1–64.6), menthone (1.4–28.1) [66]	[27]
*Maconellicoccus hirsutus*	*Citrus aurantifolia*	*Citrus*	Rutaceae	limonene (71.7), β-pinene (8.5), γ-terpinene (7.3) [67]	[27]
*Maconellicoccus hirsutus*	*Zingiber officinale*	*Zingiber*	Zingiberaceae	α-zingiberene (29.9), β-sesquiphellandrene (11.2), camphene (8.6) [68]	[27]
*Planococcus citri*	*Pimpinella anisum*	*Pimpinella*	Apiaceae	trans-anethole (91.3), trans- pseudoisoeugenyl-2-methylbutyrate (2.5), p-anisaldehyde (1.6) [69]	[36]
*Planococcus citri*	*Thymus vulgaris*	*Thymus*	Lamiaceae	p-cymene (35.96), terpinen-4-ol (10.29), α-terpinene (8.85) [20]	[36]
*Planococcus citri*	*Mentha piperita*	*Mentha*	Lamiaceae	menthol (70.08), menthone (14.49), limonene (4.32) [70]	[36]
*Planococcus citri*	*Origanum onites*	*Origanum*	Lamiaceae	carvacrol (48.0), terpinen-4-ol (6.8), sabinene hydrate (6.1) [71]	[36]
*Planococcus citri*	*Rosmarinus officinalis*	*Rosmarinus*	Lamiaceae	1,8-cineole (44.97), camphor (10.79), caryophyllene (9.43) [72]	[36]
*Phenacoccus solenopsis*	*Cinnamomum verum*	*Cinnamomum*	Lauraceae	(E) cinnamaldehyde (35.6), linalool (18.92), eugenol (18.69) [73]	[74]
*Drosicha mangiferae*	*Syzygium aromaticum*	*Syzygium*	Myrtaceae	eugenol (97.1), trans-caryophyllene (1.7) [32]	[37]
*Drosicha mangiferae*	*Cymbopogon citratus*	*Cymbopogon*	Poaceae	trans-citral (37.9), cis-citral (31,8), limonene (18.1) [32]	[37]
*Drosicha mangiferae*	*Datura alba*	*Datura*	Solanaceae	thymol (60.3), carvacrol (30.2), D-verbenone (1.0) (*Datura* genus) [52]	[37]
*Maconellicoccus hirsutus*	*Jatropha curcas*	*Jatropha*	Euphorbiaceae	δ-cadinene (9.6), α-epi-cadinol (7.4), pulegone (6.0) [75]	[76]
*Maconellicoccus hirsutus*	*Ricinus communis*	*Ricinus*	Euphorbiaceae	α-thujone (31,71), 1,8- cineole (30,98), α-pinene (16,88) [77]	[76]
*Maconellicoccus hirsutus*	*Azadirachta indica*	*Azadirachta*	Meliaceae	γ-elemene (20.8), germacrene-B (20.3), trans-caryophyllene (13.5) [78]	[79]
*Dysmicoccus brevipes*	*Citrus aurantium*	*Citrus*	Rutaceae	D-limonene (78.5), γ-terpinene (12.7), α-pinene (2.1) [47]	[47]
*Dysmicoccus brevipes*	*Citrus limon*	*Citrus*	Rutaceae	D-limonene (59.8), β-pinene (14.7), γ-terpinene (10.2)	[47]
*Dysmicoccus brevipes*	*Citrus sinensis*	*Citrus*	Rutaceae	D-limonene (83.3), linalool (8.9), myrcene (3.6)	[47]
*Planococcus ficus*	*Minthostachys verticillata*	*Minthostachys*	Lamiaceae	pulegone (57.0), menthone (36.3), isomenthone (1.7)	[26]
*Planococcus ficus*	*Eucalyptus globulus*	*Eucalyptus*	Myrtaceae	1,8-cineole (76.7), limonene (18.9), β-phellandrene (1.7)	[26]
*Pseudococcus jackbeardsleyi*	*Eupatorium odoratum*	*Eupatorium*	Asteraceae	linalool (21.64), β-pinene (9.43), 1,3-cycloheptadiene (8.92) [80]	[32]
*Pseudococcus jackbeardsleyi*	*Cinnamomum bejolghota*	*Cinnamomum*	Lauraceae	eugenol (82.05), trans-caryophyllene (3.8), 2-methoxy-4-propenylphenyl acetate (3.5) [32]	[32]
*Pseudococcus jackbeardsleyi*	*Ocimum basilicum*	*Ocimum*	Lamiaceae	linalool (43.78), eugenol (13.66) 1,8- cineole (10.18) [81]	[32]
*Pseudococcus jackbeardsleyi*	*Piper betle*	*Piper*	Lauraceae	safrole (44.25%), eugenol (5.16%), β-caryophyllene (5.98%) [82]	[32]
*Pseudococcus jackbeardsleyi*	*Eucalyptus globulus*	*Eucalyptus*	Myrtaceae	1,8-cineole (76.7), limonene (18.9), β-phellandrene (1.7) [26]	[32]
*Pseudococcus jackbeardsleyi*	*Syzygium aromaticum*	*Syzygium*	Myrtaceae	eugenol (97.1), trans-caryophyllene (1.7) [32]	[32]
*Pseudococcus jackbeardsleyi*	*Piper nigrum*	*Piper*	Piperaceae	α-bergamotene (14.57), caryophyllene (11.47), β-bourbonene (8.47) [83]	[32]
*Pseudococcus jackbeardsleyi*	*Cymbopogon citratus*	*Cymbopogon*	Poaceae	trans-citral (37.9), cis-citral (31.8), limonene (18.1) [32]	[32]
*Pseudococcus jackbeardsleyi*	*Cymbopogon nardus*	*Cymbopogon*	Poaceae	citronellal (41.7), geraniol (20.8), β-elemene (11.0) [84]	[32]
*Pseudococcus jackbeardsleyi*	*Citrus aurantifolia*	*Citrus*	Rutaceae	limonene (71.7), β-pinene (8.5), γ-terpinene (7.3) [67]	[32]
*Pseudococcus jackbeardsleyi*	*Citrus hystrix*	*Citrus*	Rutaceae	D-limonene (25.28), β-pinene (21.10), sabinene (14.99) [85]	[32]
*Pseudococcus jackbeardsleyi*	*Citrus maxima*	*Citrus*	Rutaceae	limonene (97.4), β-mycrene (1.2), α-phellandrene (0.7) [86]	[32]
*Pseudococcus jackbeardsleyi*	*Citrus reticulate*	*Citrus*	Rutaceae	limonene (91.65), γ-terpinene (6,17), β-pinene (0.93) [87]	[32]
*Pseudococcus jackbeardsleyi*	*Alpinia nigra*	*Alpinia*	Zingiberaceae	1,8-cineole (34.0), α-fenchylacetate (13.1), α-terpineol (9.6%) [88]	[32]
*Pseudococcus jackbeardsleyi*	*Amomum krervanh*	*Amomum*	Zingiberaceae	1,8-cineole (58.53), α-pinene (8.31), α-terpinyl acetate (4.68) [89]	[32]
*Pseudococcus jackbeardsleyi*	*Curcuma longa*	*Curcuma*	Zingiberaceae	α-turmerone (13.6–31.5), ar-turmerone (6.8–32.5), β-turmerone (4.8–18.4) [90]	[32]
*Pseudococcus jackbeardsleyi*	*Zingiber cassumunar*	*Zingiber*	Zingiberaceae	triquinacene,1,4-bis (methoxy) (26.5), (Z)-ocimene (22.0), terpinen-4-ol (18.5) [91]	[32]
*Pseudococcus jackbeardsleyi*	*Zingiber officinale*	*Zingiber*	Zingiberaceae	α-zingiberene (29.9), β-sesquiphellandrene (11.2), camphene (8.6) [68]	[32]
*Phenacoccus solenopsis*	*Pelargonium graveolens*	*Pelargonium*	Geraniaceae	citronellol (27.67), cis-menthone (10.23), linalool (10.05) [92]	[93]
*Phenacoccus solenopsis*	*Thymus vulgaris*	*Thymus*	Lamiaceae	p-cymene (35.96), terpinen-4-ol (10.29), α-terpinene (8.85) [20]	[93]
*Phenacoccus solenopsis*	*Cymbopogon citratus*	*Cymbopogon*	Poaceae	trans-citral (37.9), cis-citral (31.8), limonene (18.1) [32]	[93]
*Paracoccus marginatus*	*Cinnamomum multiflorum*	*Cinnamomum*	Lauraceae	Methyleugenol (49.4), cinnamaldehyde (29.6), palmitic-acid (4.2), eugenol (3.0) [25,94]	[25]

The volatile content of each EO is expressed as relative percentage (%) by peak area normalization.

## Data Availability

Not applicable.
